# Editorial: Vision in Cephalopods

**DOI:** 10.3389/fphys.2018.00018

**Published:** 2018-01-22

**Authors:** Frederike D. Hanke, Daniel C. Osorio

**Affiliations:** ^1^Institute for Biosciences, Sensory and Cognitive Ecology, University of Rostock, Rostock, Germany; ^2^School of Life Sciences, University of Sussex, Brighton, United Kingdom

**Keywords:** eye, cephalopods, visual system, camouflage, cognition, development, movement, genetics

Cephalopods' large eyes constantly scanning their environment give these fascinating animals a curious and attentive appearance; often human visitors to aquaria or divers feel that they are being watched (Darmaillacq et al.). There is a good literature on cephalopod vision, especially anatomy, learning and motor control in octopus, and on cuttlefish camouflage (see for example Young, 1960, 1962; Wells, 1978; Mather and Anderson, 1995; Kelman et al., 2008; Hanlon et al., 2011; Chiao et al., 2015; How et al.). The collection in this research topic of *Frontiers in Physiology* highlights innovative work in the field. Often methodological developments underpin advances in physiology. Here we find magnetic resonance imaging (MRI) is proving to be a valuable anatomical tool. Chung and Marshall used high resolution MRI and histology to link eye anatomy of various squid species to species-specific habitats. MRI also served to reveal changes in the three dimensional structure of cuttlefish optic lobes, first during ontogenesis, and then with the maturation of body patterning and other visuomotor behavior (Liu et al.). Innovative methods are also reported by Hadjisolomou and El-Haddad who developed a software plugin to measure size and color of multiple chromatophores mediating the cephalopods extraordinary abilities to camouflage, and by Bublitz et al. who describe a new experimental procedure to conduct behavioral experiments with octopus including the establishment of a secondary reinforcer.

Contributions united in this research topic cover a broad thematic range. Following the developmental theme explored by Liu et al., Imarazene et al. describe genes that are involved in cuttlefish eye development, while Darmaillacq et al. review literature on the development of visual function and visual learning in embryonic cuttlefish. In behavior of adult cuttlefish, Schnell et al. demonstrate lateralization of eye use during predatory and antipredatory behavior. It is fascinating to learn of similarities to vertebrate and arthropod lateralization, which support the idea that lateralization evolves to allow the animals to perform diverse tasks efficiently by allowing neural specialization.

Another group of papers within our research topic deals with vision and locomotion. Levy and Hochner elegantly describe a simple mechanism that allows *Octopus vulgaris* to control and coordinate its eight arms. It seems that octopus decides from moment to moment which arm to recruit, and usually uses the arm that is most likely to move the animal in the desired direction. For cuttlefish, Helmer et al. document a saccadic movement strategy, which might indicate that they use optic flow for distance estimation. Cuttlefish are generally bottom-dwelling animals, Scatà et al. show that they prefer to move horizontally over the ground, making detours around obstacles, moving vertically over obstacles only when this is essential, a behavioral choice which may miminize the risk of detection by predators.

And, of course, there is work on cephalopod camouflage. Cephalopods vary their appearance with unparalleled subtlety and speed by controlling the dilation of many thousands of individual chromatophores, which are innervated motoneurons that run directly from the brain. Besides the already bespoken chromatophore fine scale monitoring software plugin (Hadjisolomou and El-Haddad), Josef et al. combine two of the poorly studied questions in an ingenious study: how cuttlefish integrate information across heterogeneous environments, and how they conceal themselves when moving. They report that moving cuttlefish match a subsample of the substrate slightly larger than the body in the direction of their movement. Continuing the theme of movement, but this time in the body patterns themselves, How et al. provide a comprehensive review of the diversity of dynamic skin patterns in cephalopods, and discuss the possible function of these remarkable and enigmatic displays.

Cephalopods are often noted for their cognitive abilities. In the first of two papers on visual cognition, Lin and Chiao show that cuttlefish can classify diverse objects as visually equivalent—resembling categorical perception—and that they can recognize objects when they are partially occluded. Learning theory continues to offer an influential framework of understanding animal cognition. Bublitz et al. work in the tradition of learning experiments of the 1960s, testing reversal learning in octopus, but with methodological innovations as already mentioned. They document a high degree of individuality: some animals do not learn the reversal, whereas others learn to reverse multiple times.

The Stazione Zoologica Anton Dohrn has for nearly 150 years offered to science the pleasures of Naples and the wealth of the sea, nurturing ground-breaking discoveries in neuroscience, behavior and evolution of cephalopods, and beyond. A fascinating contribution to the theme by Dröscher offers a historical perspective on vision research at the Stazione Zoologica. Cephalopod vision research in general and on the retinal ultrastructure in particular was initiated only a few years after the foundation of the Stazione Zoologica (Grenacher, 1884) and remained in focus throughout the twentieth century nourished by the famous work of for example Boycott (Boycott et al., 1965), Young (Young, 1971), Moody, Robertson, and Pariss (Moody and Robertson, 1960; Moody and Parriss, 1961), Sutherland, Muntz, and Mackintosh (Sutherland, 1954; Sutherland and Muntz, 1959; Sutherland and Mackintoshh, 1971) as summarized by Dröscher. Dröscher's manuscript also includes a previously unpublished early twentieth century debate on color vision between the skeptical Carl von Hess and the innovative young Karl von Frisch, future winner of the Nobel Prize in Physiology.

This Research Topic emerged from a Workshop organized in Naples as satellite to the 2014 Annual Meeting of the COST Action FA1301 (http://www.cephsinaction.org/), led by Dr. Giovanna Ponte. We were appointed as Guest Editors following the meeting at the suggestion of Prof. Graziano Fiorito, coordinator of the CephsInAction Task-Force for scientific dissemination, who initially proposed the Research Topic to Frontiers in Physiology. We believe that the COST Action FA1301 has made a wonderful contribution to cephalopod science through meetings, research exchanges and education and will benefit our subject for many years to come. We are delighted to thank Dr. Ponte and Prof Fiorito for their support, and hospitality.

## Author contributions

All authors listed have made a substantial, direct and intellectual contribution to the work, and approved it for publication.

### Conflict of interest statement

The authors declare that the research was conducted in the absence of any commercial or financial relationships that could be construed as a potential conflict of interest.

## References

[B1] Boycott B. B. Lettvin J. Y. Maturana H. R. Wall P. D. (1965). Octopus' optic responses. Exp. Neurol. 12, 247–256. 14314553 10.1016/0014-4886(65)90070-1

[B2] Chiao C.-C. Chubb C. Hanlon R. T. (2015). A review of visual perception mechanisms that regulate rapid adaptive camouflage in cuttlefish. J. Comp. Physiol. A 201, 933–945. 10.1007/s00359-015-0988-5 25701389

[B3] Grenacher H. (1884). Abhandlungen zur vergleichenden Anatomie des Auges. I. Die Retina der Cephalopoden. Abhandl. Naturforsch. Ges. Halle 16, 1–50.

[B4] Hanlon R. T. Chiao C.-C. Mäthger L. M. Buresch K. C. Barbosa A. Allen J. J. (2011). Rapid adaptive camouflage in cephalopods, in Animal Camouflage, eds Stevens M. Merilaita S. (Cambridge: Cambridge University Press), 145–163.

[B5] Kelman E. J. Osorio D. Baddeley R. J. (2008). A review of cuttlefish camouflage and object recognition and evidence for depth perception. J. Exp. Biol. 211, 1757–1763. 10.1242/jeb.015149 18490391

[B6] Mather J. A. Anderson R. C. (1995). Personalities of octopuses *(Octopus rubescens)*. J. Comp. Psychol. 107, 336–340.

[B7] Moody M. F. Parriss J. R. (1961). The discrimination of polarized light by *Octopus*: a behavioural and morphological study. Zeitschr. Vergleich. Physiol. 44, 268–291.

[B8] Moody M. F. Robertson J. D. (1960). The fine structure of some retinal photoreceptors. J. Biophys. Biochem. Cytol. 7, 87–92. 14423794 10.1083/jcb.7.1.87 PMC2224851

[B9] Sutherland N. S. (1954). Figural after-effects, retinal size, and apparent size. Quart. J. Exp. Psychol. 6, 35–44.

[B10] Sutherland N. S. Mackintoshh N. J. (1971). Mechanisms of Animal Discrimination Learning. New York, NY; London: Academic Press.

[B11] Sutherland N. S. Muntz W. R. A. (1959). Simultaneous discrimination learning and preferred directions of motion in visual discrimination of shape in *Octopus vulgaris* Lamarck. Pubbl. Staz. Zool. Napoli 31, 109–126.

[B12] Wells M. J. (1978). Octopus - Physiology and Behaviour of an Advanced Invertebrate. London: Chapman and Hall Ltd.

[B13] Young J. Z. (1960). The visual system of *Octopus*. Nature 186, 836–839. 13846638 10.1038/186836a0

[B14] Young J. Z. (1962). The optic lobes of *Octopus vulgaris*. Philos. Trans. R. Soc. Biol. Charact. 245, 19–65.

[B15] Young J. Z. (1971). The Anatomy of the Nervous System of Octopus vulgaris. Oxford: Clarendon Press.

